# Incidence and risk factors of VTE in patients with cervical cancer using the Korean national health insurance data

**DOI:** 10.1038/s41598-021-87606-z

**Published:** 2021-04-13

**Authors:** Jin-Sung Yuk, Banghyun Lee, Myoung Hwan Kim, Kidong Kim, Yong-Soo Seo, Sung Ook Hwang, Yong Kyoon Cho, Yong Beom Kim

**Affiliations:** 1 Department of Obstetrics and Gynecology, Sanggye Paik Hospital, School of Medicine, Inje University, 1342, Dongil-ro, Nowon-gu, Seoul, Republic of Korea; 2 Department of Obstetrics and Gynecology, Inha University Hospital, Inha University School of Medicine, 27, Inhang-ro, Sinheung-dong, Jung-gu, Incheon, Republic of Korea; 3 Department of Obstetrics and Gynecology, Seoul National University Bundang Hospital, 82, Gumi-ro 173 beon-gil, Bundang-gu, Seongnam-Si, Gyeonggi-Do Republic of Korea

**Keywords:** Cancer, Surgical oncology

## Abstract

This study investigated incidence and risk factors for venous thromboembolism (VTE) in patients with cervical cancer. We selected 49,514 patients newly diagnosed with cervical cancer from the Korean Health Insurance Review and Assessment Service databases. During the total follow-up period and first 6 months after initiation of primary treatments, incidence of VTE, and association of risk factors with VTE occurrence were evaluated according to primary treatments or no treatment, surgery, radiotherapy, and chemotherapy. VTE occurred in 1.15% of patients with cervical cancer. Regardless of the period after initiation of primary treatments, and of VTE, the incidence of thromboembolism was highest in chemotherapy. During the first 12 months, monthly incidence of VTE was highest in chemotherapy and decreased with time in all primary treatments. Compared with no treatment, VTE risk significantly increased for all primary treatments (surgery: HR 1.492; 95% CI 1.186–1.877) (radiotherapy: HR 2.275; 95% CI 1.813–2.855) (chemotherapy: HR 4.378; 95% CI 3.095–6.193) and for chemotherapy during the first 6 months (HR 3.394; 95% CI 2.062–5.588). In this cohort study, incidence and risk of VTE in patients with cervical cancer were the highest when chemotherapy was the primary cancer treatment, and incidence of VTE decreased with time.

## Introduction

Venous thromboembolism (VTE), including deep vein thrombosis (DVT) and pulmonary embolism (PE), aggravates morbidity and mortality and increases economic burden in patients with cancer^1–3^. Although a predisposition to hypercoagulability in patients with cancer induces VTE, VTE can also be caused by a combination of acquired factors, including age, immobility, surgery, trauma, smoking, obesity, medical comorbidity, history of VTE, chemotherapy, and hormone therapy^1,4,5^.

Incidence of VTE in patients with cancer has been reported to be 20%^6^. Current guidelines recommend pharmacologic and mechanical prophylaxis and treatment of VTE in patients with cancer^7–10^. However, current guidelines do not specify different risks and managements for VTE in different cancer types. Pharmacologic thromboprophylaxis prior to chemotherapy is recommended in ambulatory cancer patients with intermediate or high risk for VTE (Khorana score 2 or higher)^7,8^. Using this score, gynecologic cancers as a group are considered high risk, although different gynecologic cancer types have heterogeneous risks for VTE^11^.

Incidence of VTE in patients with gynecologic cancer has been reported to be 0%-41.5% because of heterogeneity in the population, diagnostic methods, and study designs^12–27^. Although VTE is a common complication in gynecologic cancer that induces high morbidity and mortality, studies evaluating their relationship were relatively uncommon^1^. Therefore, current understanding and management of VTE in gynecologic cancers is mainly based on studies of solid cancers^1,7–10^.

Study of the incidence of VTE related to various risk factors in different types of gynecologic cancers evaluated using big data may guide prophylaxis and treatment of VTE according to different gynecologic cancer types. Therefore, this study aimed to investigate the incidence and risk factors for VTE in patients with cervical cancer using Korean Health Insurance Review & Assessment Service (HIRA) data.

## Results

**Figure 1:**
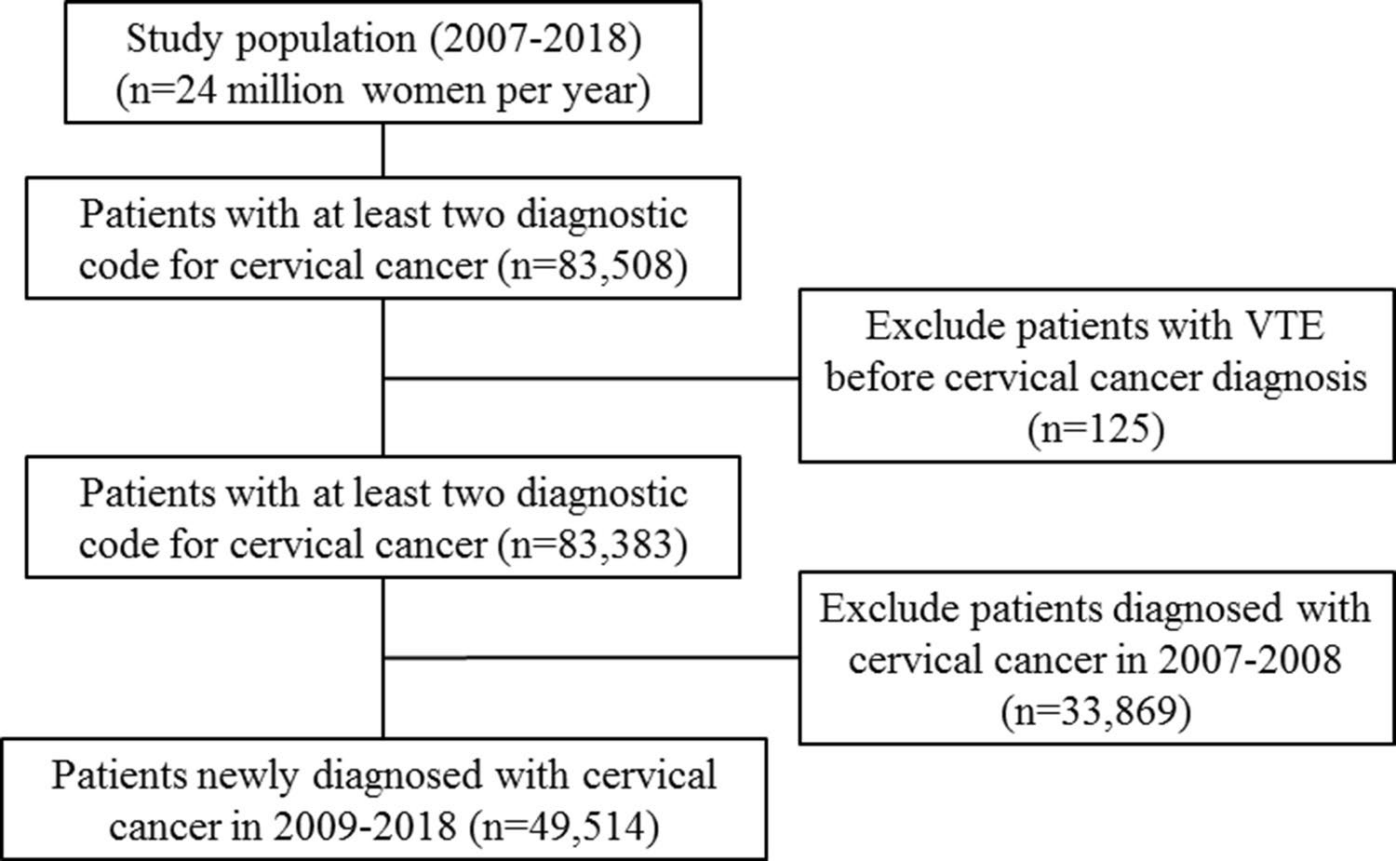
Flow chart for extracting patients included in the study. VTE, venous thromboembolism.

Data from 83,508 women who had two or more diagnostic codes for cervical cancer between 2007 and 2018 were extracted. Of these, 49,514 women newly diagnosed with cervical cancer since 2009 were selected (Fig. 1).

### Characteristics of patients with cervical cancer

Characteristics of patients with cervical cancer are shown in detail in Table 1. The mean follow-up period was 1,543.9 ± 4.9 days.

**Table 1:** Characteristics of patients with cervical cancer in HIRA claims data of 2009–2018.

	The total follow-up period				The first six months			
	VTE (-)	VTE (+)	Total	*P* value	VTE (-)	VTE (+)	Total	*P* value
	n = 48,946 (98.9%)	n = 568 (1.2%)	n = 49,514 (100%)		n = 49,293 (99.6%)	n = 221 (0.5%)	n = 49,514 (100%)	
**Mean age (years), mean** ± *SE*	53.8 ± 0.1	58.5 ± 0.5	53.9 ± 0.1	< 0.001 ^a^	53.8 ± 0.1	58.8 ± 0.9	53.9 ± 0.1	< 0.001 ^a^
**SES, n (%)**				0.165				0.838
Mid- or high-SES	45,853 (93.7)	524 (92.3)	46,377 (93.7)		46,167 (93.7)	210 (93.3)	46,377 (93.7)	
Low SES	3,093 (6.3)	44 (7.7)	3,137 (6.3)		3,122 (6.3)	15 (6.7)	3,137 (6.3)	
**CCI, n (%)**				0.008				0.095
0	26,493 (54.1)	272 (47.9)	26,765 (54.1)		26,654 (54.1)	111 (49.3)	26,765 (54.1)	
1	6,075 (12.4)	76 (13.4)	6,151 (12.4)		6,120 (12.4)	31 (13.8)	6,151 (12.4)	
2	9,380 (19.2)	111 (19.5)	9,491 (19.2)		9,449 (19.2)	42 (18.7)	9,491 (19.2)	
3	2,860 (5.8)	44 (7.7)	2,904 (5.9)		2,893 (5.9)	11 (4.9)	2,904 (5.9)	
Over 4	4,138 (8.5)	65 (11.4)	4,203 (8.5)		4,173 (8.5)	30 (13.3)	4,203 (8.5)	
**Year of cervical cancer diagnosis, n (%)**				< 0.001				0.001
2009	5,447 (11.1)	67 (11.8)	5,514 (11.1)		5,495 (11.1)	19 (8.4)	5,514 (11.1)	
2010	5,362 (11)	70 (12.3)	5,432 (11)		5,411 (11)	21 (9.3)	5,432 (11)	
2011	5,169 (10.6)	58 (10.2)	5,227 (10.6)		5,203 (10.6)	24 (10.7)	5,227 (10.6)	
2012	4,747 (9.7)	50 (8.8)	4,797 (9.7)		4,785 (9.7)	12 (5.3)	4,797 (9.7)	
2013	4,990 (10.2)	59 (10.4)	5,049 (10.2)		5,029 (10.2)	20 (8.9)	5,049 (10.2)	
2014	4,669 (9.5)	52 (9.2)	4,721 (9.5)		4,707 (9.5)	14 (6.2)	4,721 (9.5)	
2015	4,648 (9.5)	60 (10.6)	4,708 (9.5)		4,684 (9.5)	24 (10.7)	4,708 (9.5)	
2016	4,793 (9.8)	81 (14.3)	4,874 (9.8)		4,834 (9.8)	40 (30)	4,874 (9.8)	
2017	4,687 (9.6)	47 (8.3)	4,734 (9.6)		4,704 (9.5)	30 (13.3)	4,734 (9.6)	
2018	4,434 (9.1)	24 (4.2)	4,458 (9)		4,437 (9)	21 (9.3)	4,458 (9)	
**Primary treatments, n (%)**				< 0.001				< 0.001
No treatement	16,771 (34.3)	123 (21.7)	16,894 (34.1)		16,833 (34.2)	61 (27.1)	16,894 (34.1)	
Surgery	19,003 (38.8)	198 (34.9)	19,201 (38.8)		19,125 (38.8)	76 (33.8)	19,201 (38.8)	
Radiotherapy ± chemotherapy	11,477 (23.4)	203 (35.7)	11,680 (23.6)		11,613 (23.6)	67 (29.8)	11,680 (23.6)	
Chemotherapy	1,695 (3.5)	44 (7.7)	1,739 (3.5)		1,718 (3.5)	21 (9.3)	1,739 (3.5)	
**Surgery, n (%)**								
Total hysterectomy	4,761 (25.1)	25 (12.6)	4,786 (24.9)	< 0.001	4,781 (25)	5 (6.6)	4,786 (24.9)	< 0.001
Radical hysterectomy	13,872 (73)	168 (84.8)	14,040 (73.1)	< 0.001	13,971 (73.1)	69 (90.8)	14,040 (73.1)	< 0.001
Trachelectomy (radical or simple)	370 (1.9)	5 (2.5)	375 (2)	0.443 ^e^	373 (2)	2 (2.6)	375 (2)	0.662 ^b^
**Radiotherapy, n (%)**								
CCRT	4,220 (36.8)	87 (42.9)	4,307 (36.9)	0.075	4,286 (36.9)	21 (31.3)	4,307 (36.9)	0.347
EBRT	5,475 (47.7)	108 (53.2)	5,583 (47.8)	0.12	5,559 (47.9)	24 (35.8)	5,583 (47.8)	0.049
Brachytherapy	7,511 (65.4)	130 (64)	7,641 (65.4)	0.677	7,606 (65.5)	35 (52.2)	7,641 (65.4)	0.023
**Chemotherapy, n (%)**								
Platinum (cisplatin, carboplatin)	652 (38.5)	26 (59.1)	678 (39)	0.006	663 (38.6)	15 (71.4)	678 (39)	0.002
Other agents	1,038 (61.2)	18 (40.9)	1,056 (60.7)	0.006	1,050 (61.1)	6 (28.6)	1,056 (60.7)	0.002
Bevacizumab	239 (14.1)	7 (15.9)	246 (14.1)	0.734	241 (14)	5 (23.8)	246 (14.1)	0.205
Time between primary treatments and VTE diagnosis (days), mean ± *SE*		568.3 ± 30.3				37.6 ± 16.6		

*CCRT* concurrent chemoradiation therapy, *EBRT* external beam radiation therapy, *SE* standard error.

^a^ The Mann–Whitney U test was used for this analysis. ^b^The Fisher's exact test was used for this analysis.

### Incidence of VTE according to various primary treatments

VTE occurred in 1.15% and in 0.45% patients during the total follow-up period and during the first six months after initiation of primary treatments, respectively. Regardless of the period after initiation of primary treatments, VTE, DVT and PE, thromboembolism occurred in increasing order of frequency with chemotherapy, radiotherapy, surgery, and no treatment (Table 2).

**Table 2:** Incidence of VTE according to various primary treatments in patients with cervical cancer (HIRA claims data of 2009–2018).

	The total follow-up period						The first six months						Total cases
	VTE		DVT		PE		VTE		DVT		PE		
	Count	Incidence (%)	Count	Incidence (%)	Count	Incidence (%)	Count	Incidence (%)	Count	Incidence (%)	Count	Incidence (%)	
**Primary treatments**													
No treatment	123	0.73	66	0.39	65	0.38	61	0.36	32	0.19	34	0.20	16,894
Surgery	198	1.03	115	0.60	98	0.51	76	0.40	46	0.24	37	0.19	19,201
Radiotherapy	203	1.74	119	1.02	99	0.85	67	0.57	37	0.32	36	0.31	11,680
Chemotherapy	44	2.53	26	1.50	22	1.27	21	1.21	11	0.63	13	0.75	1,739
Total cases	568	1.15	326	0.66	284	0.57	225	0.45	126	0.25	120	0.24	49,514

**Figure 2:**
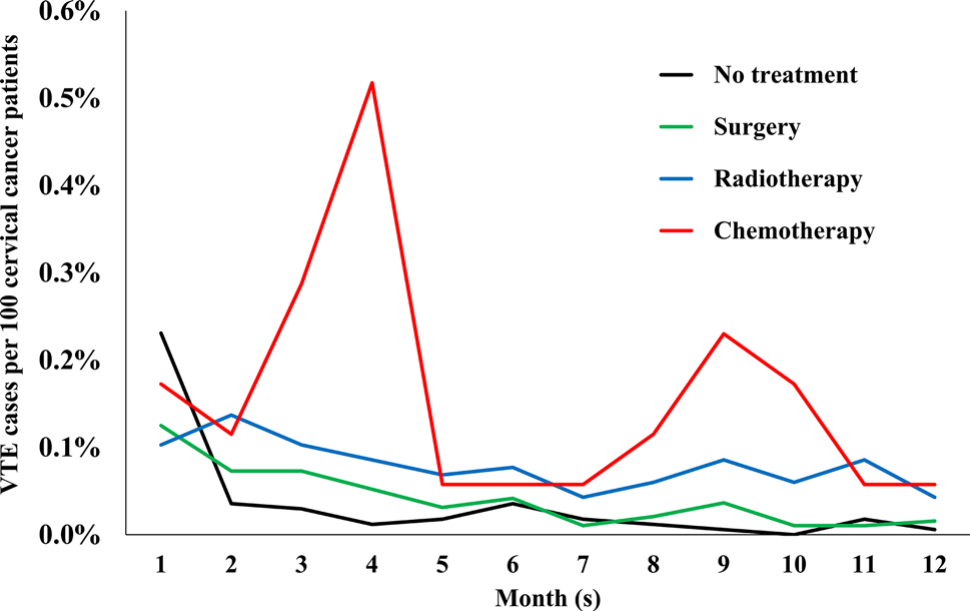
Incidence of VTE for first 12 months after initiation of primary treatment in patients with cervical cancer (HIRA claims data of 2009–2018).

During the first 12 months after initiation of primary treatments, monthly incidence of VTE occurred in increasing order of frequency with chemotherapy, radiotherapy, surgery, and no treatment, and decreased with time regardless of type of primary cancer treatment (Fig. 2).

During the total follow-up period, incidence of VTE increased with age for various primary treatments (chemotherapy, radiotherapy, and no treatment in order of frequency) and decreased after 60 years with surgery. The highest incidence occurred between 50 and 55 years for chemotherapy, between 50 and 60 years for radiotherapy, and at more than 75 years for no treatment (Supplementary Figure S1).

### Association of risk factors with VTE occurrence

During the total follow-up period, all primary treatments were associated with significant increase in VTE risk compared with no treatment when adjusted for other confounding factors (surgery: hazard ratio (HR) 1.492; 95% confidence interval (CI) 1.186–1.877; *P* = 0.001) (radiotherapy: HR 2.275; 95% CI 1.813–2.855: *P* < 0.001) (chemotherapy: HR 4.378; 95% CI 3.095–6.193: *P* < 0.001). Risk of VTE also significantly increased according to increased age, CCI, and year of cervical cancer diagnosis (age: HR 1.136; 95% CI 1.1–1.173; *P* < 0.001) (CCI: HR 1.094; 95% CI 1.052–1.138; *P* < 0.001) (year of cervical cancer diagnosis: HR 1.107; 95% CI 1.07–1.145; *P* < 0.001). Low SES was not a risk factor for VTE (Table 3).

**Table 3:** Association of risk factors with VTE occurrence in patients with cervical cancer (HIRA claims data of 2009–2018).

	The total follow-up period						The first six months					
	VTE		DVE		PE		VTE		DVE		PE	
	HR (95% CI)	*P* value	HR (95% CI)	*P* value	HR (95% CI)	*P* value	HR (95% CI)	*P* value	HR (95% CI)	*P* value	HR (95% CI)	*P* value
**Unadjusted HR**												
Age per 5 years	1.165 (1.13–1.201)	< 0.001	1.132 (1.088–1.178)	< 0.001	1.209 (1.158–1.263)	< 0.001	1.147 (1.094–1.202)	< 0.001	1.11 (1.043–1.181)	0.001	1.199 (1.122–1.28)	< 0.001
Low SES	1.383 (1.017–1.881)	0.039	1.428 (0.957–2.132)	0.081	1.446 (0.944–2.215)	0.09	1.107 (0.656–1.869)	0.704	1.336 (0.7–2.548)	0.38	0.814 (0.358–1.851)	0.624
CCI	1.136 (1.094–1.18)	< 0.001	1.155 (1.101–1.211)	< 0.001	1.091 (1.029–1.156)	0.003	1.11 (1.046–1.177)	< 0.001	1.112 (1.029–1.203)	0.008	1.063 (0.973–1.161)	0.178
Year of cervical cancer diagnosis	1.114 (1.077–1.152)	< 0.001	1.097 (1.05–1.147)	< 0.001	1.122 (1.07–1.178)	< 0.001	1.106 (1.055–1.16)	< 0.001	1.112 (1.043–1.185)	0.001	1.095 (1.026–1.168)	0.006
**Type of primary treatment**												
Surgery	1.249 (0.997–1.564)	0.053	1.349 (0.997–1.826)	0.053	1.174 (0.858–1.606)	0.316	1.022 (0.729–1.431)	0.901	1.179 (0.751–1.852)	0.473	0.893 (0.56–1.422)	0.633
Radiotherapy	2.483 (1.984–3.107)	< 0.001	2.699 (1.997–3.647)	< 0.001	2.292 (1.676–3.136)	< 0.001	1.529 (1.081–2.163)	0.017	1.612 (1.004–2.588)	0.048	1.468 (0.919–2.346)	0.108
Chemotherapy	4.279 (3.03–6.041)	< 0.001	4.687 (2.975–7.385)	< 0.001	4.024 (2.479–6.531)	< 0.001	3.389 (2.064–5.565)	< 0.001	3.391 (1.709–6.729)	< 0.001	3.74 (1.973–7.087)	< 0.001
**Adjusted HR**												
Age per 5 years	1.136 (1.1–1.173)	< 0.001	1.096 (1.05–1.143)	< 0.001	1.192 (1.138–1.249)	< 0.001	1.137 (1.081–1.196)	< 0.001	1.096 (1.025–1.172)	0.007	1.205 (1.123–1.292)	< 0.001
Low SES	1.08 (0.791–1.475)	0.627	1.152 (0.768–1.73)	0.494	1.092 (0.709–1.683)	0.69	0.885 (0.521–1.505)	0.653	1.135 (0.589–2.187)	0.705	0.611 (0.267–1.4)	0.244
CCI	1.094 (1.052–1.138)	< 0.001	1.121 (1.067–1.179)	< 0.001	1.036 (0.974–1.102)	0.262	1.063 (0.998–1.131)	0.056	1.078 (0.993–1.171)	0.073	0.996 (0.906–1.094)	0.933
Year of cervical cancer diagnosis	1.107 (1.07–1.145)	< 0.001	1.09 (1.043–1.139)	< 0.001	1.118 (1.066–1.173)	< 0.001	1.1 (1.049–1.153)	< 0.001	1.104 (1.036–1.176)	0.002	1.092 (1.023–1.165)	0.008
**Type of primary treatment**												
Surgery	1.492 (1.186–1.877)	0.001	1.607 (1.18–2.188)	0.003	1.396 (1.013–1.923)	0.042	1.233 (0.872–1.743)	0.237	1.407 (0.885–2.236)	0.149	1.063 (0.659–1.716)	0.802
Radiotherapy	2.275 (1.813–2.855)	< 0.001	2.582 (1.904–3.502)	< 0.001	1.972 (1.436–2.708)	< 0.001	1.413 (0.996–2.006)	0.053	1.539 (0.954–2.482)	0.077	1.272 (0.792–2.042)	0.32
Chemotherapy	4.378 (3.095–6.193)	< 0.001	4.653 (2.945–7.349)	< 0.001	4.351 (2.674–7.079)	< 0.001	3.394 (2.062–5.588)	< 0.001	3.312 (1.664–6.592)	0.001	3.99 (2.099–7.582)	< 0.001

*CI* confidence interval, *HR* hazard ratio.

During the first six months, only chemotherapy was associated with significant increase in VTE risk compared with no treatment when adjusted for other confounding factors (HR 3.394; 95% CI 2.062–5.588: *P* < 0.001). Risk of VTE significantly increased according to increased age and years of cervical cancer diagnosis (age: HR 1.137; 95% CI 1.081–1.196; *P* < 0.001) (year of cervical cancer diagnosis: HR 1.1; 95% CI 1.049–1.153; *P* < 0.001). Low SES and CCI were not risk factors of VTE (Table 3).

### Incidence of VTE according to pharmacologic thromboprophylaxis after various primary treatments

In this cohort, 13.1% of patients with cervical cancer received prophylactic anticoagulants. During the total follow-up period after initiation of primary treatments, 4.8% patients with VTE did not receive prophylactic anticoagulants (4.3% of DVT and 4.6% of PE) and 95.2% received prophylactic anticoagulants (95.7% of DVT and 95.4% of PE). Incidence of VTE was 0.1% (0% of DVT and 0% of PE) in patients did not receive prophylactic anticoagulants and 8.3% (4.8% of DVT and 4.2% of PE) in patients who received prophylactic anticoagulants (Supplementary Table S1).

Prescribed prophylactic anticoagulants were UFH (77.8%), DOAC (17.4%), warfarin (17.2%), LMWH (9.7%), and fondaparinux (0.0%). DOAC was the most frequently used therapeutic anticoagulant (52.6%), and 6.7% of patients with VTE used an inferior vena cava filter anticoagulants (Supplementary Table S2).

## Discussion

Incidence of VTE in patients with cervical cancer has been reported between 0.6 and 41.5%^13–16,18,20,22,24–27^. Previous studies have reported that the incidence of perioperative VTE in Asian patients was lower than that of the Western population^28^. In our study, incidence of VTE in patients with cervical cancer was lower than in previous studies, corresponding to characteristics of an Asian population^13–16,18,20,22,24–27^.

In two cohort studies that evaluated incidence of VTE within 30 days (n = 175) and 90 days (n = 4,158) from gynecologic cancer surgery, the incidence of VTE decreased with time^29,30^. In one cohort study, the absolute rate of VTE in patients with cervical cancer (n = 704) decreased with time after cancer diagnosis^31^. Corresponding to these findings, our study demonstrated that incidence of VTE decreases with time for all types of primary cancer treatments, including surgery.

Some studies reported that in patients with cervical cancer who underwent radical hysterectomy (laparoscopic or abdominal) and/or pelvic and/or para-aortic lymphadenectomy, incidence of VTE ranged between 2.7 and 9.5%^13–16,22^. In a prospective study performed in patients with gynecologic cancer (n = 411), prior pelvic radiation therapy was a risk factor for postoperative DVT^32^. Moreover, in patients receiving gynecologic brachytherapy (n = 329), incidence of VTE was 1.2%^12^. The claims database analysis of one cancer cohort (n = 17,284) showed that, in patients with common solid tumors including ovarian cancer treated with chemotherapy, the incidence of VTE was 12.6% in the 12 months after initiation of chemotherapy (1.4% in non-cancer patients matched individually (1:1) to those with cancer)^33^. Among all chemotherapeutic agents, the risk of VTE was highest in patients receiving cisplatin and bevacizumab (odds ratios: 1.36 and 1.43, respectively)^33^. A retrospective study reported that in patients with cervical cancer (n = 798), chemotherapy was a risk factor for VTE^25^. In our study, incidences and risks of VTE were high in the order of chemotherapy, radiotherapy, surgery and no treatment regardless of the time period after initiation of primary cancer treatments, VTE, DVT and PE. Patients who receive chemotherapy as initial treatment for cervical cancer have more advanced disease such as locally advanced disease and distant metastasis, and patients with advanced-stage cancer appear to be at a greater risk of VTE occurrence^22,34^. Therefore, in our study, chemotherapy as the primary therapeutic modality with the highest incidence and risk for VTE might be attributed to the advanced stage. However, determination of risk of VTE in patients by type of primary treatment might provide significant information for patients with cervical cancer.

In one cervical cancer cohort (n = 272), patients older than 60 years had a fourfold increased risk of VTE compared to patients younger than 60 years (10.4% vs. 2.6%). In another cervical cancer cohort (n = 704), the absolute rate of VTE increased in patients over 60 years (12 per 1000 person years vs. 30 per 1000 person years)^22,31^. In our study, regardless of time period after initiation of primary treatments and/or VTE (DVT and PE), patients with VTE were older than patients without VTE. In addition, risk of VTE increased with increasing age.

In our study, we assume that patients with low SES receive adequate cancer management under the Korean national health insurance system because low SES was not a risk factor for VTE. The risk of VTE increased in patients diagnosed with cervical cancer more recently, which might be attributed to developments in diagnostic techniques for VTE and improved accessibility under the Korean national health insurance system.

During the total follow-up period after initiation of primary treatments, 95.2% of patients with VTE received prophylactic anticoagulants in our study. The incidence of VTE was higher in patients who received prophylactic anticoagulants than in patients who did not receive them (8.3% vs 0.1%). We presume that, in our study, patients exposed to known risk factors of VTE, such as old age, high CCI, radical hysterectomy, radiotherapy, and chemotherapy, receive prophylactic anticoagulants more frequently compared to those without such risk factors.

The significance of this study was the investigation of the incidence and risk factors for VTE VTE in patients with cervical cancer using a large scale cohort. To our knowledge, this is the first report that identifies incidence and risk of VTE according to primary cancer treatments in patients with cervical cancer. This study has the following limitations based on the use of claims data. First, because diseases in this study were indirectly defined based on diagnostic and prescription codes without reviewing medical records, some patients may have been incorrectly diagnosed by erroneous coding. However, based on our inclusion criteria, this study might have included patients who were incorrectly diagnosed. Second, patients who did not have a prescription code or only had one prescription code for anticoagulants were not considered VTE patients. Under the Korean national health insurance system, patients diagnosed with VTE, regardless of symptoms, are usually treated for VTE. Therefore, there should have been few patients who truly did not receive treatment for VTE. Third, this study excluded patients with VTE before cervical cancer diagnosis, and patients with VTE before initiation of primary cancer treatments were not considered VTE patients. It is difficult to define eligible patients because the order of cervical cancer or VTE diagnosis and primary cancer treatment may be altered if they occur over a short time. However, criteria were used to clarify relationships between VTE and cervical cancer or primary cancer treatment. Finally, this study could not investigate relationships between VTE and body mass index or stages and/or histologic types of cervical cancer because the HIRA dataset does not provide this information. However, each primary treatment may represent a risk of VTE in patients with cervical cancer compared with a no treatment group comprised of a large population in any cancer stage, although the analysis was not adjusted for stage.

Based on a large Korean cohort study, we evaluated the incidence and risk factors of VTE and time to VTE occurrence in patients with cervical cancer. Incidence and risk of VTE were highest in patients who received chemotherapy as a primary cancer treatment, and incidence of VTE decreased with time for all primary cancer treatment types. The results of our study contribute to management of patients with cervical cancer.

## Methods

### Study population and design

South Korea has a universal health coverage system, the National Health Insurance, which covers approximately 98% of the overall Korean population^35,36^. The HIRA shares most National Health Insurance Service data. Diagnosis codes for cancer among medical information have high accuracy because patients with cancer receive additional medical payment discounts. The claims data of the HIRA represent 23 million women per year^35^. This is a retrospective cohort study using the entire insurance data from the HIRA between January 1, 2007 and December 31, 2018.

The 10th revision of the International Statistical Classification of Diseases and Related Health Problems (ICD-10), the Health Insurance Medical Care Expenses (2017 and 2018 version), and the HIRA Drug Ingredients Codes were used for diagnostic codes, surgery codes, and prescription codes to select eligible patients. Patients with cervical cancer were defined as patients who had two or more diagnostic codes for cervical cancer (ICD-10: C53x) between 2007 and 2018. Among patients diagnosed with cervical cancer, patients who had diagnostic codes for VTE (ICD-10: I80.2, I80.3, I26) before the first C53x codes were excluded. Patients who had C53x codes between 2007 and 2008 (wash out period) were also excluded to select only patients with newly diagnosed cervical cancer. Patients with VTE were defined as patients who have prescription codes for anticoagulants more than 2 times simultaneously with diagnostic codes for VTE after initiation of primary cancer treatment (surgery or radiotherapy or chemotherapy). If patients did not get primary cancer treatment (no treatment group), patients with VTE were defined as patients who have prescription codes for anticoagulants more than 2 times simultaneously with diagnostic codes for VTE from the date of the first diagnostic code for cervical cancer. Deep vein thrombosis (DVT) and pulmonary embolism (PE) were defined as patients who have prescription codes for anticoagulants more than 2 times simultaneously with diagnostic codes of I80.2 or I80.3 and I26, respectively.

Age was categorized in intervals of 5 years. Low socioeconomic status (SES) was defined as patients with medicaid as National Health Insurance. The Charlson Comorbidity Index (CCI) was calculated according to Quan's method using data between 365 days and 1 day before the first diagnostic date of cervical cancer^37^. Primary treatments (no treatment, surgery, radiotherapy, and chemotherapy) were defined as treatments performed first after diagnosis of cervical cancer. No treatment was defined as lack of prescription code for surgery, radiotherapy, and chemotherapy. Surgery was defined by surgery codes for total hysterectomy, radical hysterectomy, and trachelectomy (radical or simple) simultaneously with diagnostic codes of cervical cancer. If two or more surgeries were performed, the first surgery was used as the primary treatment. Neoadjuvant chemotherapy followed by laparotomy or laparoscopy was considered as surgery. Radiotherapy was defined as prescription codes for radiotherapy simultaneously with diagnostic codes for cervical cancer. Chemotherapy was defined as prescription codes for chemotherapy (bevacizumab, carboplatin, cisplatin, docetaxel, fluorouracil, gemcitabine, ifosfamide, irinotecan, mitomycin, paclitaxel, topotecan) simultaneously with the diagnostic code for cervical cancer. Pharmacologic thromboprophylaxis for VTE was defined as prescription for anticoagulants more than 2 times without diagnostic codes for VTE after initiation of primary treatment or from date of cervical cancer diagnosis (if treatment was not performed). Prophylactic anticoagulants comprised unfractionated heparin (UFH), low molecular weight heparin (LMWH), warfarin, fondaparinux, and direct oral anticoagulants (DOAC). Therapeutic anticoagulants comprised UFH, LMWH, warfarin, aspirin, fondaparinux, and DOAC.

Data from the total follow-up period and first 6 months after initiation of primary treatments were analyzed.

### Statistical analyses

All statistical analyses in this study were performed using SAS Enterprise Guide version 6.1 (SAS Institute, Inc., Cary, NC, USA) and R version 3.3.2 (R Foundation for Statistical Computing, Vienna, Austria). Continuous variables were analyzed using the independent t-test and Mann–Whitney U test, whereas categorical variables were analyzed using the Chi-square test and Fisher’s exact test. In addition, associations between variables and VTE occurrence were analyzed using the Cox Proportional Hazard Regression model with or without adjusting for confounding factors such as age per 5 years, low SES, CCI, year of cervical cancer diagnosis, and types of primary treatment. All statistical analyses were performed using two-tailed tests and *P* values < 0.05 were considered statistically significant. When there was a missing value, the mean imputation method was used.

### Ethics

Based on the South Korea’s Bioethics and Safety Act, because the HIRA dataset uses anonymous identification codes to protect personal information, approval of this study was waived by the Institutional Review Board of Inha University Hospital (No. 2019-11-007) on November 25, 2019 and informed consent was not required.

## Supplementary Information

Supplementary Information.

## Data Availability

The datasets generated during and/or analysed during the current study are not publicly available because the HIRA dataset restricts access to the information. Researchers can access to datasets only during analysis and take results of analysis without original datasets.
