# Supplementary material for: Incidence and risk factors of VTE in patients with cervical cancer using the Korean national health insurance data
Source: Sci Rep. 2021 Apr 13;11:8031. doi: 10.1038/s41598-021-87606-z (PMC8044206; doi:10.1038/s41598-021-87606-z)
Supplement: Supplementary file 1 — Supplementary Information. [file 41598_2021_87606_MOESM1_ESM.docx]

**Incidence and risk factors of VTE in patients with cervical cancer using the Korean national health insurance data**

Jin-Sung Yuk^1^, Banghyun Lee^2*^, Myoung Hwan Kim^1^, Kidong Kim^3^, Yong-Soo Seo^1^, Sung Ook Hwang^2^, Yong Kyoon Cho^1^, Yong Beom Kim^3^

**Supplementary Figure S1. Incidence of VTE according to age increments in patients with cervical cancer (HIRA claims data of 2009-2018).**

**
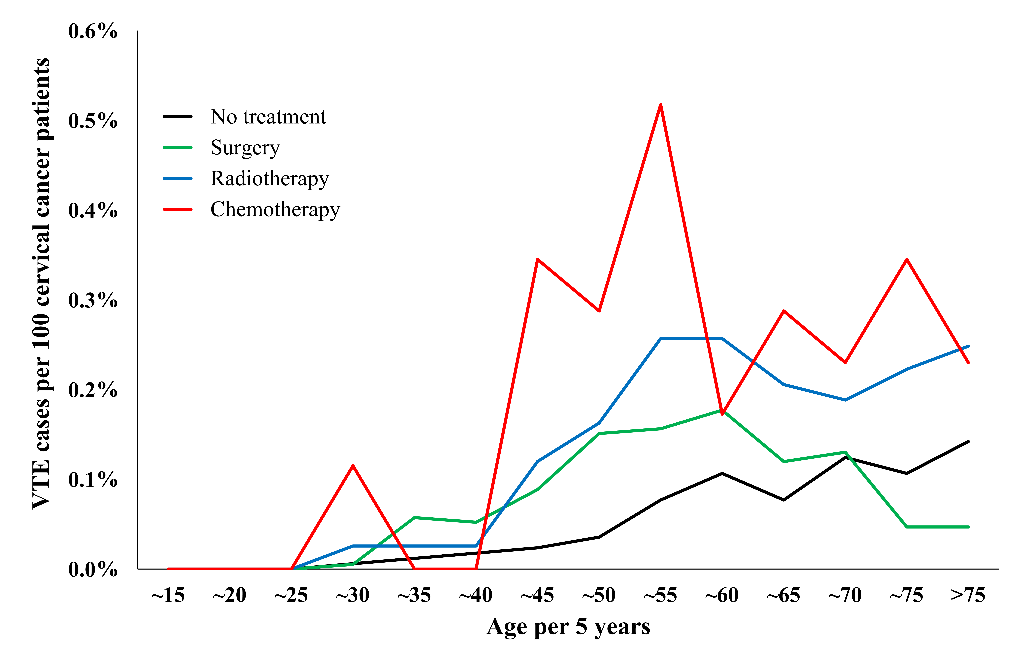
**

**Supplementary Table S1. Incidence of VTE according to pharmacologic thromboprophylaxis in patients with cervical cancer (HIRA claims data of 2009-2018).**

|  | Total | VTE | | | DVT | | | PE | | |
| --- | --- | --- | --- | --- | --- | --- | --- | --- | --- | --- |
|  | (n=49,514) | No | Yes | *P* value | No | Yes | *P* value | No | Yes | *P* value |
|  |  | (n=48,946) | (n=568) |  | (n=49,188) | (n=326) |  | (n=49,230) | (n=284) |  |
| Pharmacologic  thromboprophylaxis, n (%) |  |  |  | <0.001 |  |  | 0.001 |  |  | <0.001 |
| No | 43,009 (86.9) | 42,982 (99.9) | 27 (0.1) |  | 42,995 (100) | 14 (0) |  | 42,996 (100) | 13 (0) |  |
| Yes | 6.505 (13.1) | 5,964 (91.7) | 541 (8.3) |  | 6,193 (95.2) | 312 (4.8) |  | 6,234 (95.8) | 271 (4.2) |  |

**Supplementary Table S2. Methods for prophylaxis and treatment of VTE in patients with cervical cancer (HIRA claims data of 2009-2018).**

DOAC, direct oral anticoagulants; IVC, Inferior Vena Cava; LMWH, low molecular weight heparin; UFH, unfractionated heparin.

|  | Prophylaxis (n=6,505) | Treatment (n=568) |
| --- | --- | --- |
| UFH, n (%) | 5,060 (77.8) | 287 (50.5) |
| LMWH, n (%) | 629 (9.7) | 99 (17.4) |
| Fondaparinux, n (%) | 1 (0.0) | 0 (0) |
| Warfarin, n (%) | 1,122 (17.2) | 257 (45.3) |
| Aspirin, n (%) |  | 252 (44.4) |
| DOAC, n (%) | 1,129 (17.4) | 299 (52.6) |
| Thrombectomy, n (%) |  | 2 (0.4) |
| Thromboplasty, n (%) |  | 0 (0) |
| Thrombolysis, n (%) |  | 3 (0.5) |
| IVC filter, n (%) |  | 38 (6.7) |
